# Effects of Sudarshan KriyaYoga and Advanced Meditation Program on Genetic Expression of Pro-inflammatory and Antioxidants Genes

**DOI:** 10.7759/cureus.41377

**Published:** 2023-07-04

**Authors:** Lakshmi Bhaskar, Chhaya Kharya, Monojith Debnath, Thrinath Mullapudi, Manjula Subbanna, Deepika Chhabra, Neeta Kumar, Prem Prakash Sharma, Om Lata Bhagat, Vinod Kochupillai

**Affiliations:** 1 Sri Sri Institute for Advanced Research, Ved Vignan Maha Vidya Peeth, Bengaluru, IND; 2 Department of Human Genetics, National Institute of Mental Health and Neurosciences, Bengaluru, IND; 3 Division of Social Health Implementation, Indian Council of Medical Research, New Delhi, IND; 4 Department of Community Medicine and Family Medicine, All India Institute of Medical Sciences, Jodhpur, Jodhpur, IND; 5 Department of Physiology, All India Institute of Medical Sciences, Jodhpur, Jodhpur, IND

**Keywords:** hollow and empty meditation, antioxidants, pro-inflammatory, genetic expression, sudarshan kriya yoga

## Abstract

Background

Stress leads to immune system dysregulation and dyshomeostasis at the gene level. Mind-body practices are known to influence genomic expression, leading to better health and quality of life.

Objective

To assess the effect of Advanced Meditation Program (AMP) on the mRNA expression of pro-inflammatory and antioxidative genes among those already practicing Sudarshan Kriya Yoga (SKY).

Methods

A total of 97 healthy volunteers participated in the study, distributed into two groups. The Group I SKY practitioners attended a four-day AMP (50 participants with an average age of 38.8 ± 11.9 consisting of 37 females and 13 males); they are first-time participants of the AMP. Group II SKY practitioners, on the other hand, consisted of 47 participants with an average age of 36.4 ± 9.3 with 43 females and four males. At day 0, day 5, and day 90, the mRNA expression of pro-inflammatory genes, namely interleukin (IL) 1β, IL6, and the tumor necrosis factor (TNF), and the expression of antioxidative genes, namely superoxide dismutase (SOD), catalase, and glutathione peroxidase (GPx) was observed. The data were analysed in two phases due to the emergence of coronavirus disease 2019 (COVID-19): (i) pre-COVID-19 and (ii) during COVID-19.

Results

In the pre-COVID-19 data set, IL1β, IL6, and TNF were found to have decreased in both groups. There is a significant increase in the expression of SOD and catalase in Group I and a decrease in Group II by day 90. During COVID-19, pro-inflammatory genes increased in Group I and had no significant change in Group II. All three antioxidant genes had decreased expression by day 90 in Group I; SOD decreased in Group II.

Interpretation and conclusions

Reduced expression of pro-inflammatory genes and increase in the expression of antioxidative genes during the pre-COVID-19 time suggest that the practice of SKY and added AMP may enhance antioxidative defense and may reduce the chance of getting diseases related to inflammation in the body.

## Introduction

Acute/chronic stress related to modern, fast-paced life leads to immune system dysregulation, which offsets the balance of pro-inflammatory cytokines and antioxidant mechanisms, suppressing homeostatic functions [1]. Various mind-body practices originating from ancient Asian traditions (primarily from India) have been introduced over the last few decades to increase self-awareness and improve health and quality of life.

These include various meditations such as mindfulness, Vipassana (a mindfulness Buddhist meditation), Sahaj Samadhi (effortless blissful trans), transcendental meditation (TM), and others, breathing processes such as Sudarshan kriya, alternate nostril breathing, Kapalbhati (fast forceful exhalations), Bhramari Pranayama (bumblebee breath), yoga, and tai chi. All these practices lead to increased wellness, a calm state of mind, equanimity, improved attention, better emotion regulation, a state of relaxation, and a reduction in symptoms of chronic inflammatory diseases. Also, there is improvement in various mental issues like depression, anxiety, and emotional dysregulation [2-7]. The positive effects of mind-body activities influence the genomic expressions that are mediated via signal transduction and epigenetic mechanisms. According to Black and his team, signal transduction is the adaptive capacity for intracellular molecules to respond to extracellular signals due to changing environment [3]. Whereas Venditti et al. described that “epigenetic mechanisms represent a way to regulate gene activity in real time without modifying the DNA sequence, thus allowing the genome to adapt its functions to changing environmental contexts” [2].

There is a paucity in the literature on the impact of mind-body practices on physiological homeostatic pathways. However, modulatory effects of yoga and meditation on immune, endocrine, stress, and neural regulatory pathways have been demonstrated in the scientific literature [8]. The main focus of the research on assessing the effects of meditation practices on gene expression in immune cells has been on stress-related inflammatory markers and associated biological pathways [3,9]. Techniques such as TM, Sudarshan Kriya Yoga (SKY), and Zen meditation influence the levels of neuro-hormonal factors and impact the expression of genes and proteins [10]. Regular SKY practitioners have shown reduced blood lactate and improved antioxidant status, indicating a reduction in oxidative stress [11]. Gene expression profiling following SKY practice revealed better antioxidant status at the enzyme activity level and RNA level, along with environmental stress regulation [12]. SKY has been shown to cause rapid global gene expression alterations in peripheral blood mononuclear cells (PBMCs) immediately after the session [13]. Significant reduction in the activity of the pro-inflammatory transcription nuclear factor kappa B (NF-κB) was noted in breast cancer survivors [14] and in long-term meditators also [15]. Meditation practices lead to a level of heightened attention, awareness, and self-regulation that can mitigate stressor/threat evaluation and, thus, can quiet downstream molecular defense programs - that is, gene expression profiles [3]. Bhasin et al. observed a significant reduction in the expression of genes linked to inflammatory response and stress-related pathways, along with enhanced expression of genes associated with energy metabolism, mitochondrial function, insulin secretion, and telomere maintenance in practitioners of relaxation response (RR). These changes were significantly better among the long-term than short-term practitioners [9].

Earlier studies have suggested that different styles of meditation may have different outcomes. Most studies were conducted on groups who learned meditation using mindfulness or focused attention/concentrative methods. However, the immediate/short-term effects of guided meditations like hollow and empty meditations, those given during Advanced Meditation Program (AMP) in novices, have not been studied much. Hence, the present study was designed to assess the impact of guided meditation introduced during AMP (hollow and empty meditations) on gene expression related to inflammation and oxidative stress in regular SKY practitioners (practicing SKY for less than one year). We chose to study the mRNA expression of pro-inflammatory genes, including interleukin (IL) 1β, IL6, and tumor necrosis factor (TNF), and the expression of antioxidative genes, including superoxide dismutase (SOD), catalase, and glutathione peroxidase (GPx), among regular practitioners of SKY, before and immediately after and 90 days of the AMP.

## Materials and methods

We studied the effect of AMP in SKY practitioners from Scheduled Castes (SCs) community. SCs are those castes/races in India that have suffered from social, educational, and economic backwardness over several centuries [16]. After its independence, the Government of India enacted progressive legislation and programs for the development and empowerment of the SCs. As a result, SCs are now well-educated, well-employed, and financially well off [17]. Even though governmental policies impacted the SC community positively in terms of education and finance, attitudinal issues and a lack of self-esteem, however, have persisted in this community. Kaaren Mathias, who runs a community mental health facility in Uttarakhand (a state in north India), observed that people from SCs and Scheduled Tribes (STs) are three times more likely to be depressed compared to people from the general category [18]. Accordingly, our study group was a subset from the SC community. The goal is to provide this community with a set of evidence-based techniques to optimize their mental well-being.

Subject selection and study plan

The selection of the study’s participants was done based on a combination of convenience sampling and stratified random sampling scheme. A community of villages in Bengaluru comprising a large number of people from the SC community was identified (Saldoddi, Marathalli, Agara colony, Tataguni, Udayapura, Vasudevpura, Tharalu, Thilaknagar, Somanahalli, Lakshmipura, Kumaraswamy layout, Agara, Navagram, Girigowdanadoddi, Hosur, and Salhunsse). Bengaluru was selected based on convenience - i.e., proximity to our research lab and availability of teachers who can teach yoga and meditation in the local language. The members of our research team visited the identified SC community villages (Table 1) and conducted a door-to-door survey to assess and recruit the study participants. Those who were interested were required to attend a basic workshop of six days (two hours each day) to learn SKY. After the workshop, volunteers were informed about the study. Those who expressed interest in the AMP were put in Group I, the intervention group who underwent a four-day long AMP, during which they also regularly practiced SKY. The remaining were placed in Group II, who were required to practice SKY daily at home for about half an hour and participate in a weekly group practice under the guidance of a teacher. A total of 97 volunteers participated in the study. Fifty volunteers opted for Group I, and 47 volunteers opted for Group II. All participants completed the study. Volunteers suffering from major psychiatric, neurologic, or any other sickness such as cancer, infections in the last six weeks, cardiac disease, stroke, trauma, vascular injury to the brain, or parasitic infection such as cysticercosis or hearing impairment were excluded from the study. Ethical clearance was obtained from the Ethics Committee of Sri Sri Institute for Advanced Research, Bengaluru, India (SSIAR/IEC/05). The demographic data of both groups are shown in Table 1.

**Table 1:** Demographic details of Groups I and II AMP: Advanced Meditation Program, SKY: Sudarshan Kriya Yoga, HbA1C: hemoglobin A1C, ESR: erythrocyte sedimentation rate

	Group I (AMP + SKY)	Group II (SKY)
n -number	50	47
Age	38.8 ± 11.9	36.4 ± 9.3
Gender		
Female	37	43
Male	13	4
Education		
Not at all	5	13
Primary school	10	10
Middle School	2	2
High school	26	19
Paramedical	1	None
Degree	6	3
Marital status		
Married	37	43
Single	7	None
Living together	1	2
Separated	0	1
Widow	5	1
Physical health parameters		
Hemoglobin (g/dl)	12.67 ± 2.31	12.04±1.56
Fasting blood sugar (mg/dl)	114.15 ± 45.	106.32 ± 38.05
HbA1C (%)	6.42 ± 1.96	6.11 ± 1.41
ESR	22.02 ± 18.82	26.74 ± 18.08
Cholesterol (mg/dl)	175.94 ± 39.70	182 ± 36.1
Triglyceride (mg/dl)	158.3 ± 85.37	147.57 ± 95.76
Homocystein (cmol/l)	14.89 ± 9.51	13.16 ± 6.27
Cortisol (mg/dl)	7.96 ± 2.36	8.64 ± 4.52
Chest X-ray	All X-rays were normal	All X-rays were normal

Experimental protocol

To assess the gene expression of pro-inflammatory and antioxidative genes, a blood sample (5 ml) was collected at three time points: day 0, day 5, and day 90 for both groups. For Group I, day 0 was the baseline before the start of the AMP. Samples were also collected at day 5 (after the of completion of AMP) and at day 90 - i.e., 90 days, calculated from the baseline. Similarly, for Group II, i.e., the SKY group, blood samples were collected on day 0 (at the time of recruitment), day 5, and day 90.

Details of SKY and AMP

Details related to SKY methodology are available in earlier publications [19,20]. The SKY method includes three stages: slow Ujjayi Pranayama; Bhastrika Pranayama (fast breathing); chanting of “OM”; and rhythmic, cyclical breathing (Sudarshan kriya). The whole process is called Sudarshan Kriya Yoga (SKY).

The AMP is a four-day residential guided meditation program, also known as the silence program. This program is regularly offered and conducted at the Art of Living (AOL) center in Bengaluru, India, by trained teachers. Participants are provided accommodation, breakfast, lunch, and dinner to avoid confounders and they maintain silence for three days. The program includes morning sadhana (spiritual practice), where volunteers practice yoga postures for physical resilience, followed by SKY. They are given guided meditation sessions twice a day, from 10:00 AM to 1:00 PM and after lunch break from 3:00 PM to 5:30 PM. All the guided meditations were given through audio instructions in the voice of Gurudev Sri Sri Ravi Shankar. Meditations are called hollow and empty meditations as these meditations make participants experience hollowness and emptiness within. Each morning, participants spent one hour in some seva (service) activity like cutting vegetables, serving food, and cleaning. In the evening, they listened to mantras sung by experienced singers in the center. Group I practiced SKY and guided meditations during the residential AMP. On the other hand, Group II continued practicing SKY daily at home and came for weekly follow-up sessions.

Gene expression method

Gene expression study was carried out in the Department of Human Genetics, National Institute of Mental Health and Neuro-Sciences (NIMHANS), Bengaluru. mRNA expression of pro-inflammatory cytokines IL1β, IL6, TNF, and antioxidants SOD, catalase, and GPx was studied using the 2-ΔΔCt method of relative quantification [21]. PBMCs were separated by Ficoll-Paque density gradient centrifugation (Ficoll®-Paque Premium GE17-5442-02; Cytiva, Marlborough, MA, USA) and used for RNA isolation using a commercial spin column method (Qiagen, Inc., Limburg, Netherlands). The obtained RNA was quantified by spectrophotometry (MultiskanTM GO Microplate Spectrophotometer; Thermo Fisher Scientific Inc., Waltham, MA, USA). The RNA quality was checked by measuring 260/230 and 260/280 optical density ratios. The extracted RNA was reverse transcribed into single-stranded cDNA using high-capacity cDNA Reverse Transcription Kit (Applied Biosystems, Waltham, MA, USA). Target gene mRNA expression of IL-Iβ (Assay ID: Hs01555410), IL6 (Assay ID: Hs00985639), TNF (Assay ID: Hs00174128), SOD2 (Assay ID: Hs00167309), catalase (Assay ID: Hs00156308), and GPx (Assay ID: Hs00989766) were normalized with 18S rRNA (Assay ID: Hs03003631). The assay for every sample was run in triplicate, including the calibrator and a no-template control. Quantitative PCR (qPCR) was performed on Quantstudio 6 Real-Time PCR System (Applied Biosystems) in a 96-well format. Reaction was in a final volume of 10 μL containing TaqMan Universal PCR Master Mix, probes, and primers for target and endogenous control gene with the PCR conditions of two minutes at 50°C, 10 minutes at 95°C, followed by 40 cycles of 15 seconds at 95°C and one minute at 60°C. Results were normalized to endogenous control with target gene fold change relative to the reference sample.

Data analysis

The data collection for the study started in June 2019 and was completed by June 2021 (total duration: 25 months), with 97 volunteers who participated (Group I: 50 and Group II: 47). From March 2020 onward, COVID-19 commenced throughout the world, including India. Therefore, nearly half of the data collection inevitably happened during the COVID-19 pandemic (March 2020 to June 2021). There was a huge variation in the parameters from March 2020 onward. Therefore, data is subdivided into two parts for analysis: (i) pre-COVID-19 (Group I: 21, Group II: 26) and (ii) during COVID-19 (Group I: 29, Group II: 21).

Statistical analysis was done using GraphPad Prism version 5.0 (GraphPad Software, San Diego, CA, USA). Data were screened for the assumption of normality using parametric tests. Statistical tests were applied to compare all parameters within and between the groups. Wilcoxon signed-rank test was applied to compare parameters within the groups (day 0 and day 5, day 0 and day 90). The overall p-value was taken as significant at equal to or less than 0.05.

## Results

A total of 97 volunteers participated in the study: (i) pre-COVID-19 (Group I: 21, Group II: 26) and (ii) during COVID-19 (Group I: 29, Group II: 21). It is noticeable that except for one subject who developed COVID-19 in 2021 and succumbed to it, the remaining 96 volunteers, despite very high pro-inflammatory markers, had no signs of COVID-19 infection. Whether this was due to the practice of SKY and/or AMP is difficult to conclude from the present study.

Expression of pro-inflammatory genes

In the pre-COVID-19 phase, all three pro-inflammatory genes (IL1β, IL6, and TNF) decreased to a statistically significant extent in both the groups by day 90; TNF, however, temporarily increased in Group II by day 5. During the COVID-19 phase, IL1β and IL6 increased by day 90 in Group I, while there was no significant change in Group II. TNF temporarily decreased by day 5 in Group II, but there was no observable change by day 90 in either group (Table 2).

**Table 2:** Results of gene expression of pro-inflammatory genes in Group I and Group II Results of gene expression of pro-inflammatory genes. In the pre-COVID-19 phase, IL1β and IL6 significantly decreased in both the groups from day 0 to day 90. TNF decreased significantly in Group I at day 5 and day 90, while in Group II significantly decreased at day 90. During the COVID-19 phase, both IL1β and IL6 increased significantly at day 90 in Group I and showed no significant change in Group II. At day 90, both groups showed no significant change in TNF, whereas in Group II, it decreased significantly on day 5. (* statistically significant value.) IL: interleukin, TNF: tumor necrosis factor

Genes	Groups	Day-0		Day-5	Day-90	Day (0-5) p-value	Day (0-90) p-value
Pre-COVID-19 phase							
IL1 β	Group I		3.95 (0.15-14.91)	3.0 (0.52-12.18)	0.29 (0.1-1.35)	0.254	0.011*
	Group II	4.37 (1.66-40.5)		2.26 (0.70-89.95)	0.20 (0.05-12.04)	0.11	0.004*
IL6	Group I	3.09 (1.12-11.17)		6.85 (2.98-19.35)	1.34 (0.48-8.54)	0.22	0.014*
	Group II	2.22 (1.13-17.44)		5.05 (2.60-13.51)	0.5 (0.25-2.76)	0.288	0.001*
TNF-α	Group I	4.37 (1.27-9.93)		0.98 (0.15-2.16)	0.79 (0.11-1.66)	0.007*	0.016*
	Group II	0.97 (0.26-2.03)		1.58 (0.22-2.84)	0.18 (0.05-0.53)	0.06	0.007*
During COVID-19 phase							
IL1 β	Group I	141.4 (19.33-320.8)		177.2 (50.15-438.3)	520.8 (70.93-910.2)	0.125	0.007*
	Group II	628 (62.84-1664)		239.1 (82.06-1276)	227 (119.5-1455)	0.37	0.289
IL6	Group I	8.16 (4.64-20.01)		12.52 (6.93-24.22)	16.26 (4.34-34.15)	0.067	0.038*
	Group II	29.4 (12.81-86.36)		31.6 (17.87-129.9)	24.48 (16.6-181.1)	0.176	0.18
TNF-α	Group I	18.65 (3.19-61-48)		15.43 (5.73-45.87)	28.1 (5.80-55.31)	0.354	0.266
	Group II	42.79 (11.24-142.2)		7.27 (4.04-68.72)	22.19 (8.28-158.2)	0.017*	0.265

Expression of antioxidant genes

In the pre-COVID-19 phase, the expression of SOD and catalase increased significantly by day 90, and GPx showed no significant change in Group I. In Group II, all three (SOD, catalase, GPx) showed a decrease by day 90. During the COVID-19 phase, all three antioxidant genes had decreased expression by day 90 in Group I; SOD decreased in Group II as well, but catalase and GPx showed no significant change (Table 3).

**Table 3:** Results of gene expression of antioxidative genes in Group I and Group II Results of gene expression of antioxidative genes. In the pre-COVID-19 phase, SOD increased significantly from day 0 to day 90 in Group I, but in Group II, it decreased by day 90. Similarly, catalase increased significantly at day 90 in Group I, and in Group II, it decreased significantly at day 5 and day 90. GPx decreased significantly in Group II at day 5 and day 90, but there was no significant change in Group I. During the COVID-19 phase, SOD decreased significantly in Group I at day 90 and no significant change in Group II over 90 days. Catalase and GPx decreased significantly in Group I. Catalase and GPx showed no significant change in Group II. (* statistically significant value.) SOD: superoxide dismutase, GPx: glutathione peroxidase

Genes	Groups	Day-0		Day-5	Day-90	Day (0-5) p-value	Day (0-90) p-value
Pre-COVID 19 phase							
SOD	Group I		0.72 (0.47-1.03)	0.97 (0.79-1.17)	1.33 (0.75-1.88)	0.001*	0.006*
	Group II	0.851 (0.68-1.49		1.09 (0.67-1.61)	0.69 (0.51-0.89)	0.328	0.002*
Catalase	Group I	0.77 (0.49-1.38)		0.69 (0.61-1.63	1.54 (0.86-2.93)	0.254	0.0009*
	Group II	1.76 (1.24-2.41)		1.14 (0.87-1.88)	0.96 (0.69-1.74)	0.009*	0.09*
GPx	Group I	2.28 (0.44-3.39)		1.708 (0.51-2.98)	1.076 (0.31-3.40)	0.192	0.417
	Group II	3.03 (2.50-3.60)		2.29 (1.47-3.18)	0.49 (0.31-2.93)	0.0005*	0.0001*
During-COVID 19 phase							
SOD	Group I	2.58 (1.76-5.89)		2.48 (2.21-3.71)	2.19 (1.61-3.94)	0.484	0.012*
	Group II	5.42 (2.54-10.17)		2.77 (1.75-10.19)	5.06 (2.44-22.39)	0.489	0.078
Catalase	Group I	9.22 (3.35-18.16)		3.67 (2.18-9.71)	1.76 (0.38-5.43)	0.013*	0.0001*
	Group II	4.88 (2.43-9.03)		4.45 (1.38-6.0)	3.29 (1.92-9.87)	0.242	0.486
GPX	Group I	1.08 (0.81-2.44)		1.20 (0.81-1.62)	0.62 (0.37-0.92)	0.422	0.0002*
	Group II	1.80 (1.22-6.73)		1.35 (0.72-3.05)	1.30 (0.84-7.43)	0.103	0.445

## Discussion

The studied population was a cohort of persons from the same geographical area, caste, and economic background and were all approached at similar time points. They were trained for SKY practice and consented to performing it every day. Those in Group I (50 subjects) underwent four days of AMP in addition to practicing SKY. Accidentally, or incidentally, the current study has become unique because, while this study was being conducted, the COVID-19 pandemic emerged unexpectedly. Any pandemic, like COVID-19, was not anticipated in the process of making the study design or planning the objectives. The study was initiated in January 2019, and in March 2020, COVID-19 made an unexpected entry, affecting the whole world, including this underprivileged, remotely living rural community in India. When test results were being analyzed, a large change in values was observed at around March 2020, when the first COVID-19-related lockdowns started. Accordingly, we decided to analyze the data separately from initiation to February 2020 (pre-COVID-19) and from March 2020 to the end of the study (during COVID-19) on June 21, 2021.

Different yogic, meditative, and other mind-body practices that generate relaxation have been shown to influence the expression of various genes. To illustrate, the expression of pro-inflammatory genes reduces while antioxidative gene expression increases following such practices in general [2,3,22]. In the present research, in the pre-COVID-19 phase, pro-inflammatory gene expression decreased in both the groups, i.e., Group I (SKY + AMP) and II (SKY), indicating a statistically significant reduction in stress. Though there was a significant decrease in the expression of all three genes, i.e., IL1β, IL6, and TNF, expression of IL6 declined more in Group II, and TNF declined more in Group I. Scientific literature has revealed variable results. For instance, Cahn et al. found an increase in the level of anti-inflammatory cytokine IL10 and decrease in the pro-inflammatory cytokine IL12 after a three-month yoga and meditation retreat [8]. However, the plasma levels of other pro-inflammatory cytokines increased after the retreat, including interferon gamma, TNF, IL1β, IL6, and IL18. Scientific studies on the use of mind-body practices have also shown variable results with regard to pro-inflammatory markers/gene expression [4,23]. Expression of antioxidant genes increased in Group I, indicating better antioxidative defense in those who underwent AMP in addition to daily SKY practice. Among antioxidant genes, expression of SOD and catalase increased, but GPx showed no significant change in Group I. Moreover, in Group II, all expressions showed a decrease rather than an increase. Increased SOD and catalase and no change in GPx have also been reported among tai chi practitioners [4]. The rise in GPx after prolonged intense exercise, reported by Brites et al., is possibly due to its presence in muscle cells [24]. Thus, the results from the pre-COVID-19 phase indicate that SKY may have anti-inflammatory and antioxidant effects that are enhanced with hollow and empty guided meditation. These findings are consistent with a previously published study [12].

The data obtained for the during-COVID-19 phase showed higher values in the levels of pro-inflammatory cytokines (IL1β, IL6, and TNF) at day 0, along with an increase in the expression of antioxidative genes (SOD, catalase, and GPx) in comparison to the pre-COVID-19 values (refer to Tables 2, 3). Due to the onslaught of the pandemic, an increase in the expression of antioxidative genes at day 0 would probably reflect a response against increased oxidative stress induced by increased inflammatory processes [25]. The effect of lockdown on various aspects of life, including COVID-19-induced oxidative stress and its relationship with the severity of disease and multi-organ failure, has been published [26]. The current study, however, suggests that the COVID-19 pandemic might have influenced inflammatory/oxidative stress even when participants had no symptoms related to COVID-19. The literature review indicated that changes in the social environment (such as the experience of threat or fear via psychological processes) that trigger neural and endocrine responses, such as activation of the sympathetic nervous system and modulation of transcription factor activity, may influence human gene expression [3,27].

Inspiratory muscle training is effective as a rehabilitation strategy during recovery from COVID-19 [28]. Similar to the inspiratory muscle training device, SKY may work as a respiratory training exercise which is a rhythmic cyclic breathing technique with inspiratory resistance training in the form of ujjayi breath. Zope et al. [22] have recommended SKY as a breath of hope during COVID-19 due to its increased efficacy in improving immunity and mental and systemic health. There is a possibility, though, that no definite conclusion can be drawn that the regular practice of SKY and/or AMP might have prevented participants from getting the symptoms of COVID-19 despite the marked increase in pro-inflammatory cytokines. In connection to this, Chandran et al.’s published paper is noteworthy as it revealed robust activation of the immune system following an advanced inner engineering meditation retreat [29]. The above observations suggest that SKY may be beneficial in maintaining well-being in health and disease. Moreover, its effectiveness enhances with the addition of a hollow and empty meditation program.

## Conclusions

AMP, along with the daily practice of SKY, during normal life (pre-COVID-19 phase) reduced mRNA expression of IL1β, IL6, and TNF and increased mRNA expression of SOD, catalase, and GPx. This finding indicates an enhanced anti-inflammatory and antioxidant response. With the emergence of COVID-19 during the study, it became a challenge for the study to get consistent and conclusive results. The marked increase in the expression of pro-inflammatory genes and antioxidative genes were observed during the COVID-19 phase without any symptom and is a new finding that has emerged in the current study (which needs to be looked at in detail). This can be attributed to regular practice of SKY. In summary, our study suggests that the regular practice of SKY helps us in dealing with stressors in a better way; moreover, the incorporation of hollow and empty meditation improves the responses, making the person resilient.
